# The importance of intonation for children’s understanding of verbal irony

**DOI:** 10.3389/fpsyg.2025.1672104

**Published:** 2025-09-11

**Authors:** Jordanna Smith, Melanie Glenwright

**Affiliations:** Department of Psychology, University of Manitoba, Winnipeg, MB, Canada

**Keywords:** sarcasm, prosody, task demands, cognitive, linguistic, cross-linguistic

## Abstract

Verbal irony refers to any utterance in which the speaker’s words mean something different from their intended meaning (e.g., “You’re really on top of things” said to a disorganized person). For children who are just learning to recognize verbal irony, a crucial cue to the ironist’s intended meaning is their intonation. In this narrative review, we describe research methods for examining how intonation influences children’s understanding of verbal irony and the task demands researchers need to consider when designing these studies. Next, we examine how children weigh different cues to verbal irony as they grow older, and we identify cross-linguistic factors that can impact children’s use of intonation for irony comprehension. We offer suggestions for planning future studies on this topic by stressing the importance of reducing task demands, acoustically analyzing directional frequency changes, examining children’s intonation consideration in languages other than English, and comparing across tonal and non-tonal languages.

## 1 Introduction

When a teacher comments “*Fantastic weather we are having*” as she looks out the classroom window at an increasingly worrisome blizzard, how will her Kindergarten students interpret this message? Although these 5-year-old children are just learning to recognize verbal irony, they can rely on various cues to discern the teacher’s intended meaning, even though it is different from her literal message. These cues include the fact that the teacher’s utterance clearly contradicts the context (e.g., it is snowing heavily), the children’s world knowledge (e.g., a blizzard cannot be “fantastic” from a child’s point of view), and the speaker’s ironic tone of voice. Here, the teacher’s intonation can help children in this age group to recognize her use of verbal irony. In this narrative review paper, we provide an overview of research methods used to study children’s use of intonation as a cue to verbal irony, and we examine how research design choices can impact task demands on children. Next, we review findings on how children’s consideration of intonation and relevant cues to verbal irony changes with development. Finally, we provide an analysis of how these cues might differ between languages, and we suggest directions for future research in this field.

Verbal irony encompasses a broad category of figurative language in which the speaker’s words do not directly convey their intended meaning. Sarcasm is one form of verbal irony where the speaker uses nonliteral language to convey a critical attitude toward a person, object, or situation (Kreuz and Glucksberg, 1989). However, there is disagreement in the literature regarding the definitions of verbal irony and sarcasm, which has likely contributed to inconsistencies in findings (Burgers et al., 2011). As much of the research concerning children’s understanding of sarcasm has referred to sarcasm as verbal irony, we will do the same to maintain clarity in this paper.

Research with adult participants has established that speakers use verbal irony to criticize, to tease, to mock, and to be funny (Jorgensen, 1996). For the social functions of verbal irony to be served, the listener must recognize the irony and accurately interpret the speaker’s intended meaning. This is why ironic speakers usually provide the listener with some paralinguistic cues such as an ironic intonation, exaggerated body language (e.g., eye rolling), and expressions of mirth (e.g., laughing). Compared to a native English speaker’s usual sincere prosody, an ironic intonation tends to be slower, lower in pitch, and louder (Rockwell, 2000). Although intonation is one of the most studied cues for children’s irony understanding, this body of research has produced mixed results. These divergent findings are likely influenced by differences in theoretical assumptions about verbal irony, differences in research questions, differences in research methods and the demands posed by various methods, and differences in sociocultural functions and implications of verbal irony.

**Figure 1:**
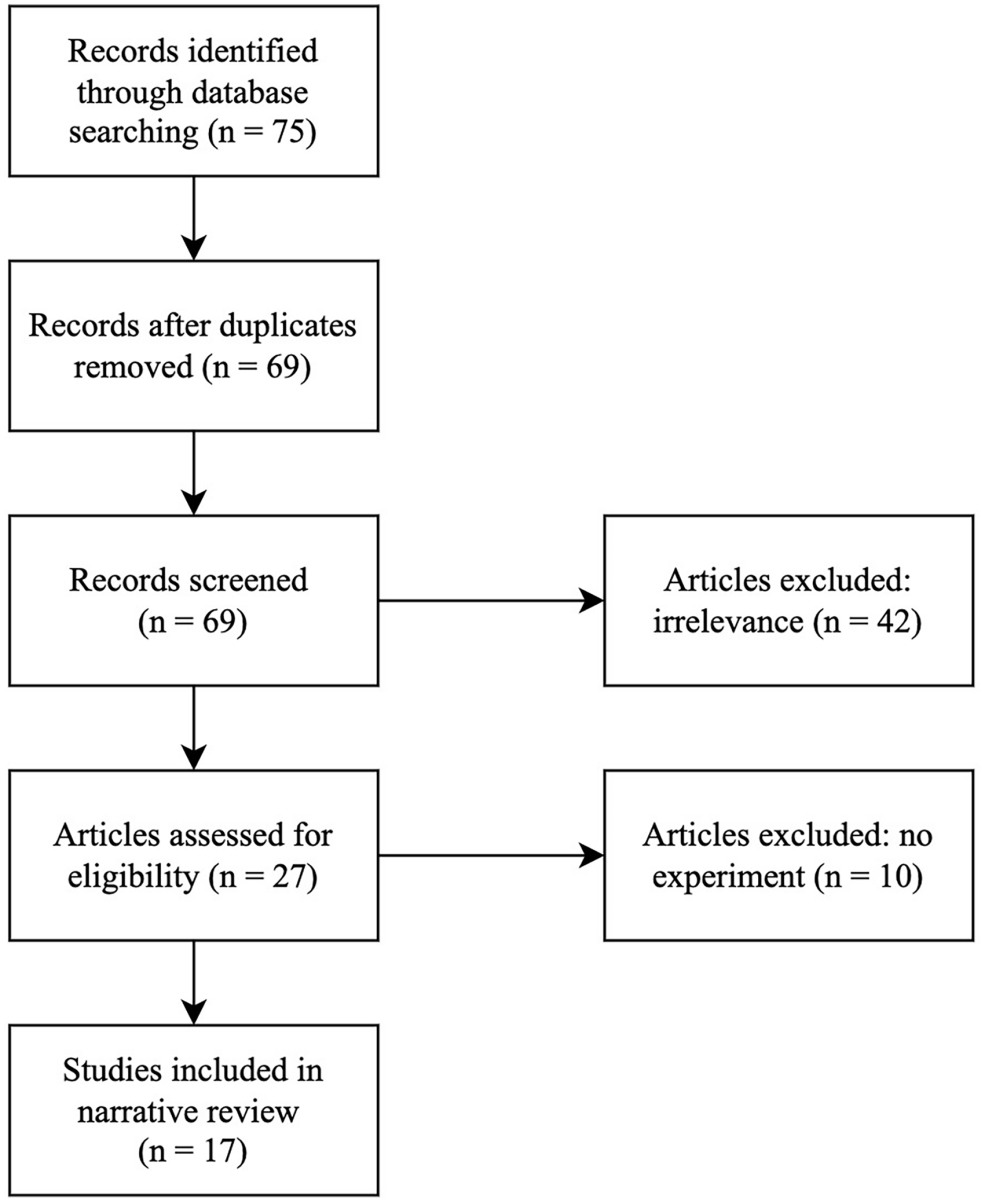
Narrative review search strategy. Articles were excluded and deemed irrelevant if the abstract did not mention examining children’s attention to ironic intonation as a key variable of interest or if the paper was not published in English. Articles were also excluded if they did not describe an experiment.

When collecting articles for this review, we searched Google Scholar and our university library database system, which include the following databases: Web of Science, PsycARTICLES, PsycINFO, Scopus, Research Gate, and Directory of Open Access Journals. We included experimental papers that had the following terms in the title or abstract: children, sarcasm or verbal irony, intonation or prosody or tone of voice. Papers were excluded if they did not describe an experiment, if the abstract did not mention examining children’s attention to ironic intonation as a key variable of interest, or if the paper was not published in English (i.e., irrelevant; see Figure 1).

### 1.1 Verbal irony theories

Many of the methodological differences discussed in this narrative review arise from differing theoretical assumptions about the nature of verbal irony. In the first study to examine children’s use of ironic intonation as a cue, Ackerman (1983) tested an informal theory suggesting that both contextual incongruity and ironic intonation are cues that signal verbal irony. He manipulated these cues to see which more strongly enhanced children’s comprehension. Children aged 6–8 years heard remarks that were either contextually congruent or incongruent, and that were delivered with either an ironic/stressed intonation or a neutral/unstressed intonation. Ackerman predicted that children would understand verbal irony better when it was spoken with ironic intonation and when the statement conflicted with the surrounding context. Since this influential paper, many researchers have adopted a similar approach - manipulating cues to verbal irony to assess their relative effectiveness in improving children’s comprehension.

Although several verbal irony theories developed for adults have been tested with children, only the echoic mention theory (Wilson and Sperber, 2012) has been used to examine how children consider intonation as a cue to verbal irony. According to this theory, listeners identify verbal irony when they detect both a critical attitude from the speaker and an “echo” of a previously expressed comment, thought, social norm, or cultural expectation. Researchers have tested this theory’s predictions by comparing children’s comprehension of verbal irony across experimental conditions that vary in the presence or absence of ironic intonation (Keenan and Quigley, 1999), explicit echoes of prior statements (Keenan and Quigley, 1999), or implicit references to a thought, social norm, or cultural expectation (Köder and Falkum, 2021). These studies collectively contribute to understanding the specific cues children use to recognize verbal irony and how those cues interact in shaping comprehension.

Other verbal irony theories have been adapted to study children’s comprehension of irony in ways that do not focus on ironic intonation. The allusional pretense theory (Kumon-Nakamura et al., 1995) proposes that ironic remarks, whether implicit or explicit, refer to failed expectations by violating norms of pragmatic sincerity. According to this theory, speakers use irony to highlight the gap between an unmet expectation and their actual attitude. Researchers have tested this theory by investigating whether children understand ironic criticisms better when they are explicitly incongruent with expectations than when they are implicitly incongruent with social norms (Hancock et al., 2000). They have also examined whether children can detect the insincerity of an ironic speaker who refers to someone else’s violation of a socially expected behavior (Creusere, 2000).

Cognitive pragmatics theory (Bara, 2010) proposes that communication follows an interaction pattern, or “behavioral game,” mutually understood by speaker and listener. Comprehension requires the listener to recognize this shared expectation. Simple communicative acts directly reference the game, whereas complex acts involve multiple, conflicting mental representations. The theory predicts a developmental sequence: children first understand and produce sincere statements (i.e., no mental conflict), then lies (i.e., one level of conflict), and finally verbal irony (i.e., multiple conflicts, making it the most difficult). Bosco et al. (2013) found that children under 7 years of age struggled with irony in both simple and complex scenarios. They concluded that irony comprehension demands more advanced inferencing about speaker intent than lies or sincere statements, emerging around 8 years of age Bosco et al. (2013) suggested that their findings support the theory’s stepwise model, with sincere statements acquired first, followed by lies, and lastly irony as the most complex form.

The Parallel Constraint Satisfaction (PCS) framework (Pexman, 2008) views irony comprehension as a rapid, simultaneous integration of multiple cues as speech unfolds. For example, if you spill coffee on yourself and a friend says with ironic intonation, *“You’re so graceful,”* PCS treats understanding this remark as an ambiguity resolution process. Cues such as the negative event outcome (e.g., spilled coffee), positive statement valence (e.g., *“You’re so graceful”*), and ironic intonation are all activated in the cognitive system, which weighs their relevance until a stable interpretation—literal compliment, ironic criticism, or white lie—emerges. According to the PCS account, a child given these cues should correctly identify the remark as ironic. This has been tested by showing children response objects representing literal (e.g., a nice duck) or ironic (e.g., a mean shark) interpretations and tracking their eye movements in real time (Climie and Pexman, 2008). Gaze direction and duration toward each object reveal how quickly children consider the speaker’s intent as nice or mean. PCS predicts early gaze shifts toward the ironic interpretation. Studies with children aged 5–8 show support for this model, demonstrating that they use cues such as the speaker’s personality traits (Climie and Pexman, 2008) and the speaker–listener relationship (Whalen et al., 2020) when interpreting irony. The PCS framework has motivated widespread use of online, time-sensitive measures to capture how children process irony in real time, not just their final interpretations.

Many of the methodological differences highlighted in this narrative review stem from divergent theoretical assumptions about what verbal irony is, how it is processed, and which cognitive and social abilities are required for its comprehension. These differing assumptions influence the design of research tasks, the measures used to assess understanding, and the interpretation of findings.

### 1.2 Nature and function of different ironic intonations

In studying the influence of intonation on children’s verbal irony comprehension, an operational definition of ironic intonation is crucial to make valid and generalizable claims. Research done with English-speaking adults suggests that ironic intonation has certain acoustic features that can be verified with a computerized signal editor. Some researchers argue that relative to sincere statements in English, ironic statements tend to be voiced slower, louder, and with a lower pitch (Rockwell, 2000; Cheang and Pell, 2008). However, research done with English-speaking children has characterized the ironic intonation in a variety of ways. Some have described their narrator’s ironic remarks as having a high pitch, strong energy, and slow rate (Milosky and Ford, 1997), or a negative, mocking, and nasal tone (Dews et al., 1996). Others have described this ironic intonation as a mocking and contemptuous tone (Winner et al., 1987), an exaggerated and mocking tone (Keenan and Quigley, 1999), or a sarcastic and mocking tone (Capelli et al., 1990).

Despite these various conceptualizations of ironic intonation, only a few studies have acoustically analyzed intonational stimuli with software (e.g., Milosky and Ford, 1997; Laval and Bert-Erboul, 2005; Köder and Falkum, 2021). For example, Glenwright et al. (2013) compared different levels of intonation by adjusting the pitch or fundamental frequency (F0) in which the ironic and literal statements were presented to English-speaking participants. For both 5- to 6-year-old children and adults, utterances with large F0 reductions including both ironic and literal statements were perceived as meaner than those with medium and small F0 reductions. However, given that intonation did not influence humor ratings for either age group (Glenwright et al., 2013), future research should explore what kinds of intonation can cue children and adults to an ironic speaker’s humorous intent.

In addition to examining the acoustic features of ironic intonation, the Glenwright et al. (2013) study represents another focus in this area of research: the specific functions served by different intonations. Specifically, prosody has been shown to influence children’s inferences of the ironic speaker’s intent or emotion. For instance, Dews et al. (1996) asked 5- to 9-year-old English-speaking children to rate various ironic utterances and found that those vocalized in a sincere (i.e., described as positive and pleasant) tone were rated mean less often and funny more often compared to utterances voiced in a deadpan (i.e., described as flat) or ironic (i.e., described as negative, mocking, and nasal) tone. The researchers concluded that a sincere intonation serves the purposes of muting the intensity and increasing the humor of the criticism, whereas a deadpan intonation makes the ironic comment seem meaner (Dews et al., 1996). Furthermore, Milosky and Ford (1997) asked adult participants to rate the prosody of various pre-recorded utterances on a 7-point scale ranging from *complimentary* to *sarcastic*. Using these stimuli, the researchers found that relative to an ambiguous intonation (i.e., rated at the midpoint of the scale 85–90% of the time) or a complimentary prosody, an ironic prosody increased the likelihood of English-speaking 6- to 9-year-old children inferring that a negative event had occurred (Milosky and Ford, 1997). These findings suggest that children can distinguish types of intonation to infer the speaker’s attitude.

Although the studies described all highlight the importance of intonation as a cue to children’s verbal irony understanding, these inconsistencies in conceptualizing and defining ironic intonation have likely contributed to the mixed findings reviewed in the present paper. It is thus important that, when planning future studies, researchers verify their stimuli with acoustical analyses to allow for subsequent replications and comparisons between studies.

### 1.3 Research methods for studying children’s consideration of ironic intonation

When examining children’s use of intonation as a cue to verbal irony, researchers have manipulated whether ironic remarks are voiced with an ironic, neutral or ambiguous intonation to assess the influence of this change on children’s interpretation of the irony. Aside from cases in which researchers analyzed the acoustic features of their ironic intonation with a computerized signal editor (e.g., Milosky and Ford, 1997; Laval and Bert-Erboul, 2005; Glenwright et al., 2013; Köder and Falkum, 2021), experimenters often verify that their narrator’s intonation sounds ironic by having adults perceptually judge their stimuli before collecting data from children (e.g., Milosky and Ford, 1997; Keenan and Quigley, 1999).

Although intonation is one of the most important cues to verbal irony for children, it rarely works in isolation and is often supplemented by other cues. Thus, most studies examining ironic intonation also incorporate some other type of cue to verbal irony, such as contextual information, violation of norms, or the ironic speaker’s facial expressions. Sometimes children are presented with visual cues such as illustrations, pictures, or puppet show props to highlight whether the speaker’s remark is congruent or incongruent with story context.

In most research described in this narrative review, story narrations were pre-recorded, and children were tested individually. These studies have used standardized methods such as presenting children with audiotaped stories, computerized delivery of stories with pre-recorded audio and photos or illustrations, videos of puppet shows, or videos of actors in conversation. These stories or conversations typically depict a scenario in which an unexpected and negative event occurs (e.g., Chris spills his juice; Zajączkowska, 2016). A speaker then produces an ironic remark that is incongruent with the scenario and voiced in an ironic intonation, such as “Well done!” (Zajączkowska, 2016). As a control condition, children are additionally shown stories where a positive event goes as expected (e.g., Chris is very careful and does not spill his juice) followed by a speaker producing a literal compliment or literal statement that is congruent with the scenario and voiced in a sincere, positive and pleasant intonation (e.g., “Well done!”; Zajączkowska, 2016). The story narration and final ironic or literal remark are usually pre-recorded by the same speaker to maintain consistency of intonation across participants and scenarios.

After each scenario is presented, researchers determine children’s comprehension of each remark by asking them questions about the facts of the story, the speaker’s belief, the speaker’s emotions, or the speaker’s intended meaning. Children’s appreciation of each remark can also be assessed by their responses to questions about the speaker’s attitude and the speaker’s intentions. After an ironic sentence (or literal sentence) has been provided, children can answer test questions by verbalizing their responses, by writing them, or by pointing to markers on rating scales. Additionally, some researchers have examined children’s online responses as the ironic sentence unfolds in real-time by recording and timing their spontaneous eye gazes toward illustrations on a computer screen. Next, we will examine the task demands of these design features and how they can influence children’s successful irony comprehension.

## 2 Task demands in methods assessing children’s verbal irony comprehension

When designing studies on children’s consideration of intonation as a cue to verbal irony, researchers must be cognizant that participating in this research poses cognitive processing demands on children. Each design choice—how verbal irony is presented, how questions are asked, and how children are required to respond—can raise or lower the threshold at which children successfully comprehend verbal irony. Researchers should aim to minimize this threshold by making design choices that place low demands on children’s attention, working memory, world knowledge, and linguistic skills.

### 2.1 Verbal irony presentation modes

When aiming to choose methods for presenting verbal irony with low task demands, researchers should maintain children’s attention and provide them with visual aids. An ideal method with low demands on attentional resources is to have children participate in the context of the ironic event so they can see a live presentation of the ironic speaker’s intonation and facial expression (e.g., Rattray and Tolmie, 2008). If this is not feasible, providing children with moving puppet shows or videos of moving people or puppets can maintain their attention better than still illustrations (Hill et al., 2016). Importantly, stories presented with illustrations are less cognitively demanding with stories presented without illustrations. Illustrations allow children to see the story events, so they are not required to construct internal representations of these events (Milosavljevic, 2024). To minimize cognitive demands, these visualizations should be simple and contain only relevant parts of the story (Köder and Falkum, 2021). Illustrations or visual aids can help children focus their attention on the stimuli, and they can provide information about the context of the ironic statement. Visual aids can also reduce linguistic demands on children by providing them with images for words that might not be in their vocabulary. If visual aids can remain in the child’s sight when they are required to answer offline test questions after the story has completed, this can help them remember the story context and the context valence with reduced demands on their working memory. Engaging methods with visual aids can increase the availability of children’s cognitive resources for processing and responding to test questions, thereby reducing the threshold for children’s verbal irony comprehension.

Researchers should also use simple language in devising verbal irony stories and aim to keep the task duration short as possible. These stories should be constructed with a basic vocabulary using short sentences to minimize linguistic demands (Banasik-Jemielniak and Bokus, 2019). It is also recommended that these stories are constructed with three or four sentences to keep the working memory demands low (Milosavljevic, 2024). Lastly, the task duration should be short (Banasik-Jemielniak and Bokus, 2019) because children can experience cognitive fatigue when they participate in several successive experimental trials without taking a rest, and this can increase their threshold for verbal irony comprehension. Next, we will consider how the format of test questions influences task demands for children.

### 2.2 Question formats

Researchers also need to consider the cognitive load of the test questions they choose. Verbal irony test questions usually refer to the speaker’s belief or intentions to gage children’s comprehension and appreciation. To answer these questions correctly, the child must process the test question, story context, and verbal irony cues to infer the speaker’s mental state and then maintain all this information in working memory while formulating a response. Shorter questions with fewer response options pose lower task demands. For example, Köder and Falkum (2021) asked children forced-choice questions with binary single-word response options (e.g., How is Mum feeling inside? Is she happy or angry?). Other researchers have asked children longer questions such as “Show me how funny or serious Jasmine was trying to be when she said, “*You picked a great spot for hide-and-seek*” then provided children with a 6-point face scale (Glenwright et al., 2013). Here, the syntactic structure of this lengthy question can pose linguistic and working memory demands on children (Fuchs, 2023), and this increases the threshold for children’s verbal irony comprehension. Compared to short options, lengthy response options for forced-choice questions require children to maintain more information in working memory to formulate a response. Furthermore, responding to several questions before answering a key comprehension question increases working memory demands (Falkum and Köder, 2024), and this can increase the threshold for children’s irony comprehension.

On the other hand, forced-choice questions can be problematic when they present children with options they would not generate or deem suitable, leading them to rely on elimination strategies rather than genuine understanding (Cassels and Birch, 2014). For instance, asking “Is [the speaker] happy or angry?” may be insufficient in cases where the speaker is expressing more subtle emotions than happiness or anger (e.g., disappointment about the weather). Children may select a response option despite feeling unsure, and in turn their forced-choice response options may not be a clear measure of their understanding. This reduces their ecological validity compared to open-ended questions, which better approximate natural conversation (e.g., “Why do you think they said that?”; Zajączkowska, 2016). Assessing verbal irony often requires children to draw on their understanding of language, infer the speaker’s beliefs or intentions, and articulate responses that reflect pragmatic awareness and metacognitive reasoning. Evidence indicates that 4- to 8-year-olds demonstrate more advanced perspective-taking and emotion recognition when assessed with open-ended than with forced-choice formats (Cassels and Birch, 2014), suggesting that open-ended questions might also be a more sensitive measure of verbal irony comprehension.

In summary, when aiming to optimize children’s success on verbal irony tasks, researchers should avoid offering children long response options because they increase the threshold for irony comprehension. Rather, they should aim to choose short forced-choice questions with minimal, carefully tailored response options or short open-ended questions. These methods reduce the risk of misinterpretation and can provide clearer insight into children’s irony comprehension processes.

### 2.3 Response modes

Verbal irony task demands also vary according to how children are required to respond to test questions. Most studies examining intonation as a cue to verbal irony have required children to respond verbally (e.g., Winner et al., 1987; Milosky and Ford, 1997; Keenan and Quigley, 1999; Rattray and Tolmie, 2008; Zajączkowska, 2016; Yu et al., 2024). When presented with these test questions, children younger than 5 years of age have difficulty formulating verbal responses that reflect their own thoughts and judgments because their structural language skills and vocabularies are limited (Milosavljevic, 2024). Questions requiring verbal responses place demands on children’s verbal explanatory skills and metapragmatic reasoning (Falkum and Köder, 2024), and this can increase the threshold for their ability to produce correct responses.

Less commonly, researchers have used questions where children could choose to respond verbally or nonverbally with a motor response by circling an icon in writing or by pointing to an icon (e.g., Dews et al., 1996; Glenwright et al., 2013). For example, some researchers have presented children with icons on a printed rating scale or a computer touchscreen, and asked children a forced-choice question such as “How funny was the turtle being? Circle one of the faces” (Dews et al., 1996). Because verbal forced-choice and open-ended responses rely on verbal ability (Cassels and Birch, 2014), using icons (e.g., emojis) as nonverbal response options can reduce the linguistic task demands compared to questions requiring a verbal response, in turn lowering the threshold for correct responses.

It is also important for researchers to recognize that their selection of response options can influence children’s verbal irony comprehension and the specificity of study results. Both the type of response icons (e.g., faces, animals, etc.) and their labels (e.g., nice, mean, angry, funny, serious, etc.) affect how well children understand the task. For example, Glenwright et al. (2013) showed 5–6-year-olds puppet show videos containing ironic criticisms, literal criticisms, and literal compliments. To assess their understanding of the speaker’s attitude, children were asked, *“Show me how nice or mean Jasmine was trying to be when she said, ‘You picked a great spot for hide-and-seek.’”* Responses were given on a 6-point Nice/Mean Scale with illustrated faces ranging from “very nice” (i.e., a joyful expression, relaxed eyebrows, broad smile) to “very mean” (i.e., furrowed brows, downturned mouth). To a 5-year-old, these faces might simply represent “very happy” to “very angry,” regardless of the provided labels. This can cause confusion, leading children to choose faces they like, or to base their choice on their own feelings or those of the addressee puppet, rather than the speaker’s intended attitude. Furthermore, labels like nice or mean may oversimplify the intended meaning—in this case, the speaker’s attitude might be more accurately described as critical or dismissive. This example shows that tailoring response-option icons and labels to children’s abilities can reduce measurement specificity and mask performance on verbal irony tasks, producing results that reflect how children interpret the options rather than their actual understanding.

All question formats described so far are offline questions where children are required to allow the experimenter to finish verbalizing the question before formulating a response. More recently, experimenters have been examining children’s spontaneous reactions to verbal irony stories as they unfold in real time. Köder and Falkum (2021) tracked children’s eye gaze locations and durations as they were presented with stories ending with ironic or literal statements that varied according to intonation and social norm information. Children’s implicit eye gaze behavior showed they were considering the ironic icon online as they heard an ironic criticism spoken, before the test question was asked. This contrasted with 6-year-old children’s explicit nonverbal pointing responses to an offline question, which indicated irony comprehension levels below a chance rate. In this study, the offline question was a direct, elicited-response question, and thus demanded a response. To answer this question correctly, a child must process the story context and verbal irony cues to infer the speaker’s mental state, maintain this information in working memory, process the test question, and formulate a response (Glenwright et al., 2021). However, when Köder and Falkum (2021) examined 3-year-old children’s online eye gaze reactions to ironic criticisms before a test question was asked, they discovered that children’s spontaneous responses could reveal some recognition of the features of verbal irony (i.e., intonation and contextual cues) that was not accounted for by the elicited-response measures. Infant cognition researchers contend that this occurs because elicited-response tasks pose considerable cognitive and verbal demands whereas spontaneous-response tasks do not (Scott and Baillargeon, 2009). These kinds of online measures assess children’s cognitive processes through their spontaneous eye gaze reactions, in turn revealing their real-time processing of a scene as it unfolds without the need to invoke a response-selection process (Scott and Baillargeon, 2009; Glenwright et al., 2021). The contrast between 3-year-olds’ spontaneous eye gazes toward the correct ironic icon and their below-chance pointing responses to offline questions in Köder and Falkum (2021) supports Scott and Baillargeon’s response-selection account. However, this account has not yet been directly tested in verbal irony research, presenting an avenue for future investigation. Nonetheless, online spontaneous-response measures such as eye-tracking methods pose significantly lower cognitive and linguistic task demands than offline elicited-response measures and can thus provide additional insight into the cognitive processes that occur when children as young as 3 interpret verbal irony.

We encourage researchers to conduct more online studies on children’s spontaneous attention to intonation as a cue to verbal irony. In the next section, we describe how children’s ability to consider cues to verbal irony changes throughout development.

## 3 Development of children’s use of intonation and other cues to understand verbal irony

Most researchers agree that children typically detect verbal irony by 6 years of age (de Groot et al., 1995; Dews et al., 1996; Fuchs, 2023; Pexman, 2023; Olkoniemi et al., 2025). However, some studies have reported recognition of some of the features of verbal irony in children as young as 3 years old (Rattray and Tolmie, 2008; Köder and Falkum, 2021). For instance, Rattray and Tolmie (2008) found that English-speaking 3-year-old children can recognize an ironic intonation, however, 4-year-olds are able to integrate a variety of cues to ironic intent (Rattray and Tolmie, 2008). Similarly, Köder and Falkum (2021) found that Norwegian-speaking 3-year-olds’ eye movements show sensitivity to some of verbal irony’s features. Relative to a deadpan (i.e., flat, slower tempo, lower pitch level, and greater intensity) intonation, a parodic (i.e., imitative and exaggerated) tone of voice led children to look more at an angry emoticon at the offset of an ironic utterance (Köder and Falkum, 2021). The authors argue that the exaggerated nature of the parodic intonation makes it easier for children to recognize the speaker’s negative attitude (Köder and Falkum, 2021). However, when asked about the speaker’s meaning after the utterance was processed, 3-year-olds incorrectly interpreted ironic utterances literally. These results suggest that this early sensitivity to intonation cues is not sufficient for successful interpretation of verbal irony (Köder and Falkum, 2021).

Despite these findings showing early recognition of intonation cues, the ability to effectively use these cues for verbal irony interpretation seems to develop a couple of years later. Some researchers have argued that by age 6, children’s interpretation of verbal irony is improved when it is uttered with an ironic intonation (de Groot et al., 1995; Zajączkowska, 2016), while others have argued that children this age are able to use intonation cues as effectively as adults (Dews et al., 1996; Nakassis and Snedeker, 2002). However, other researchers have suggested that the ability to recognize and interpret intonation cues to verbal irony continues to improve as children age beyond 6 years old. For instance, Milosky and Ford (1997) found that English-speaking 6-year-olds had more difficulty identifying ironic intonation from an ambiguous intonation than from a complimentary (i.e., to the statement, as rated by participants) intonation. This suggests that although they were able to recognize non-literal intent signaled by intonation, it was not always sufficient for correct interpretation of the irony. Furthermore, Ackerman (1983) found that English-speaking 8- to 9-year-olds were better than 6- to 7-year-olds at using intonation and context to recognize verbal irony, noting that the older children’s use of intonation cues was comparable to adults (Ackerman, 1983). Indeed, there are mixed results regarding development of the ability to use intonation as a cue to irony.

### 3.1 Children’s use of ironic intonation relative to other cues

There is also a lack of consensus regarding the order in which children recognize different cues to verbal irony. In particular, contextual incongruity cues, which are based on situational information that does not align with the literal form of the ironic statement, have received much attention in the literature (e.g., Ackerman, 1983; Winner et al., 1987; Capelli et al., 1990). Some researchers argue that children first recognize intonation cues to verbal irony, and the ability to appreciate contextual cues follows shortly after. For instance, Zajączkowska (2016) found that Polish-speaking 5-year-old children rely on intonation when interpreting irony, as contextual discrepancy cues alone are not sufficient. However, by 6 years old, children seem to rely more on contextual cues as their ability to interpret irony develops (Zajączkowska, 2016). Similarly, Laval and Bert-Erboul (2005) found that some level of understanding emerges around age 5 in French-speaking children, but only when ironic intonation was present. By 7 years old, contextual cues were equally important as intonation. This was shown by the 7-year-olds’ strategic use of both cues, which involved basing their interpretation on one cue when the other was lacking, and their hindered performance when both cues were lacking. These researchers posit that children rely more heavily on intonation at first and develop a more encompassing use of various cues to understand irony as they develop (Laval and Bert-Erboul, 2005). Other researchers hold that this primary reliance on intonation cues continues into middle childhood. Ackerman (1983) reported that English-speaking 8- to 9- year-olds use intonation as effectively as adults, but they are less sensitive to contextual discrepancy cues. He suggested that children might rely on intonation as a cue prior to developing information processing skills that allow them to integrate contextual information across sentences (Ackerman, 1983). Similarly, Capelli et al. (1990) found that 8- to 12-year-old English-speaking children rely much more heavily on ironic intonation than context in irony interpretation.

In contrast to findings highlighting children’s preference for intonation cues, there are reports that children rely more on context than intonation to understand verbal irony. For example, Stiles and Nadler (2013) compared consideration of cues in 5- to 9-year-old English-speaking children with hearing loss and with normal hearing. They found that children with hearing loss interpreted utterances as ironic and generally relied on intonation less often than children with normal hearing. However, they found that children in both groups relied more heavily on context than intonation (Stiles and Nadler, 2013), suggesting that children as young as 5 rely more on contextual cues than on intonation cues. Other researchers have taken this argument even further by claiming that 8-year-old English-speaking (Winner et al., 1987) and French-speaking (Aguert et al., 2010) children do not take intonation into account when interpreting speaker intentions. Clearly, there is a lack of consensus in the literature on the developmental sequence in children’s recognition of intonation and contextual cues to verbal irony.

Although contextual cues have received the most attention in the literature, some researchers have examined how other types of verbal irony cues work in conjunction with intonation cues. For instance, echoic mention refers to knowledge that the listener has from previous statements made by the speaker. One study examining echoic mention found increased comprehension in Norwegian-speaking 4- to 5-year-old children when a moral norm, as opposed to a social norm, was violated (Köder and Falkum, 2021). The researchers suggest that the children’s preference for the echoic mention cues may indicate that for children, intonation is less reliable than other cues in verbal irony comprehension. Similarly, Keenan and Quigley (1999) found that 6- to 10-year-old English-speaking children’s understanding of irony is best when various sources of information are available (i.e., echoic mention and ironic intonation), but that echoic mention alone can be used as a cue when necessary. Keenan and Quigley (1999) claimed that, despite the utility of intonation, it is not a necessary feature of verbal irony. In addition to echoic mention cues, researchers have identified facial expressions such as expressive mouth movements and eyebrow flashes as cues to verbal irony (Aguert, 2021). Yu et al. (2024) found that 3- to 7-year-old Mandarin-speaking children’s comprehension of ironic criticisms was improved with facial expression and intonation cues. Although intonation is often a useful cue to children learning to understand verbal irony, it is most effective when used in conjunction with various other cues such as facial expressions or echoic references to previous utterances by the ironic speaker.

Research on children’s use of cues to ironic intent has produced wildly divergent results which are likely due to diversity in research questions, research methods, and the task demands of these methods. It is also important to note that studies on children’s verbal irony comprehension across different languages reveal sociocultural differences that shape how children interpret irony (discussed in section 4.2). Next, we describe how the ironic intonation differs across languages, and we offer suggestions for how these differences might impact children’s understanding of verbal irony.

## 4 Cross-linguistic differences in ironic intonation

In the studies we have reviewed, children were presented with an ironic intonation in English, Cantonese, French, Norwegian, Mandarin, or Polish. Research with adults has demonstrated that the acoustic features of the ironic intonation vary across languages (Bettelli et al., 2024). In this section, we will review these features and suggest how these features might influence children’s understanding of verbal irony.

### 4.1 Differences in ironic intonation due to linguistic structure

One intonational feature of verbal irony that may differ between languages is the pitch variation used to signal ironic intent (Rockwell, 2000; Glenwright et al., 2013; Bettelli et al., 2024). For example, relative to literal speech, verbal irony is often spoken with a higher pitch in French, Italian, and Cantonese, whereas it is often spoken in a lower pitch in English and German (Bettelli et al., 2024). In line with this argument, studies have shown that English-speaking children and adults are more likely to recognize verbal irony when the speaker uses a lower pitch as measured by reduced F0 (Cheang and Pell, 2008; Glenwright et al., 2013). Furthermore, González-Fuente et al. (2016) tested the effect of pitch variation on French-speaking adults’ verbal irony comprehension and found that ironic utterances were produced with a higher F0 than literal utterances. In support of Bettelli et al. (2024) argument, some results show the opposite pattern for English speakers (Glenwright et al., 2013).

In addition to pitch variation, the pitch range used to signal verbal irony may also function different across languages. For instance, verbal irony spoken in languages including English, German, and Cantonese is often produced within a reduced pitch range (i.e., relative to literal speech), whereas the pitch range is generally expanded for verbal irony in languages such as French and Italian (Bettelli et al., 2024). However, this does not mean that pitch range variation is necessarily prioritized equally across languages. For instance, González-Fuente et al. (2016) found that while F0 change was a significant predictor of ironic intent in both English and French, word lengthening was a more powerful cue to verbal irony than pitch range expansion for French speakers. These findings may have important implications regarding the cues recognized by French-speaking children relative to their English-speaking counterparts, as they may rely more on rate of speech than pitch range in identifying irony. However, one similarity noted by Bettelli et al. (2024) across all six of the languages they examined (i.e., Italian, French, German, Dutch, English, and Cantonese) is the rate of speech: ironic utterances tend to have a longer duration than literal speech. This suggests that although English- and French- speakers may prioritize intonational features (i.e., pitch range, rate of speech) differently, children learning to interpret verbal irony in both languages rely on these features to some extent.

In addition to the varying features of ironic intonation across different languages, there are languages in which intonation is less flexible and thus may be more difficult for children to use as a cue to irony. For instance, 60–70% of languages (e.g., many Asian, African, and Indigenous American languages; Best, 2019) use lexical tones to distinguish words with different meanings. Although they function uniquely between different languages, these lexical tones generally restrict the pitch variation that an ironic speaker can use, since the tonal change itself alters the meaning of words. Li et al. (2013) demonstrated this constraint in a study of 8- to 12-year-old Cantonese-speaking children with and without ASD. These researchers incorporated intonation and sentence-final particles (SFPs), a linguistic feature of Cantonese that do not have a direct counterpart in English and can be used as a cue to verbal irony (Li et al., 2013). Interestingly, their results showed that for typically developing children, intonation alone was not sufficient for irony comprehension. Rather, performance was best in the other two conditions: SFPs only, and both intonation and SFPs. Li et al. (2013) argued that the discrepancy between their findings and findings from English-speaking participants (e.g., Ackerman, 1983; Capelli et al., 1990) is likely due to the tonal nature of Cantonese. The authors suggest this could be due to a mutual compensation in which greater use of sentence particles lessens the significance of intonation patterns in a language, and vice versa (Li et al., 2013). Thus, intonation is likely less prioritized as a cue for children who speak tonal as opposed to non-tonal languages. This argument is further supported by the finding that Mandarin-speaking children integrate facial expression with intonation cues for verbal irony interpretation (Yu et al., 2024), a strategy that may result from restrictions on lexical tones in Mandarin. However, the researchers incorporated this additional cue in consideration of the children with hearing loss in this study, who likely rely more heavily on non-prosodic cues than children with typical hearing.

We recommend that future studies expand on the current literature highlighting the different features of ironic intonation across languages. Furthermore, more research is necessary regarding the differences in cues used by children to recognize verbal irony in tonal and non-tonal languages. This would allow for a better understanding of specific language features that children attend to at different developmental stages.

### 4.2 Differences in ironic intonation due to cultural variation

Some researchers have argued that despite the social-cognitive processes involved in verbal irony use and comprehension, socio-cultural context is often neglected in studies on verbal irony (Banasik-Jemielniak and Kałowski, 2022). One of the most important factors in irony use and comprehension is shared knowledge between the ironic speaker and the listener, often resulting from similar experiences or attitudes (Averbeck and Hample, 2008). This implies that verbal irony may be used to serve different social functions depending on the socio-cultural context. For instance, cultures differ in their use of non-literal language, such that individualistic cultures generally prioritize direct communication while collectivistic cultures tend to value indirect meaning (Holtgraves, 1997). However, some findings have shown that people from individualistic cultures are more likely to use verbal irony than those from collectivistic cultures (Rockwell and Theriot, 2001), suggesting that verbal irony may be serving different social functions in different cultural contexts. One study examined irony perceptions across participants from three countries varying in levels of individualism/collectivism and power distance (i.e., societal emphasis placed on hierarchy): China (i.e., high collectivism and high power distance), the United States (i.e., high individualism, low power distance) and Mexico (i.e., in between China and the United States; Blasko et al., 2021). The researchers found higher rates of verbal irony use in participants from the United States and Mexico than from Chinese participants, explaining that verbal irony may pose higher social risks in China due to social dynamics of harmony and respect in power hierarchies (Blasko et al., 2021).

In consideration of the cross-linguistic variation of the social functions served by intonation, it is important to recognize that more than one ironic tone of voice has been identified for English, which is likely the case for other languages as well. These include a pretense-based, enthusiastic intonation and a more contemptuous, deadpan one (Sperber, 1984). Indeed, some studies have shown the influence of irony spoken with different types of ironic intonation on verbal irony comprehension in both 5- to 9-year-old children and adults (Dews et al., 1996). Specifically, an ironic (i.e., negative, mocking) tone seems to imply that the speaker is annoyed, whereas a deadpan (i.e., flat) intonation implies playfulness or superiority (Dews et al., 1996). In contrast, a study done with Norwegian-speaking children reported no significant differences in irony processing between utterances spoken in parodic and deadpan tones (Köder and Falkum, 2021). However, Köder and Falkum (2021) did report that children were more likely to look at an angry emoticon at the offset of an ironic utterance spoken in a parodic (i.e., relative to deadpan) tone. These findings suggest that both English- and Norwegian-speaking children use intonation cues to interpret a speaker’s attitude, even if the verbal irony is not consistently and accurately understood. Furthermore, Aguert et al. (2010) suggest that for French-speaking children, a speaker’s intonation can provide cues to both their ironic intent and their emotional state.

Clearly, there are social implications of various ironic intonations that influence children’s interpretations of verbal irony and likely vary across languages. However, due to the limited number of studies on ironic intonation in languages besides English, more research is needed to provide a clearer picture of its cross-linguistic effects on children’s understanding of verbal irony.

## 5 Directions for future research

In this section, we will synthesize suggestions for future experimental investigations of children’s consideration of ironic intonation. Milosky and Ford (1997) argued that irony comprehension depends on many different cues, and their relative strengths are determined by the specific situation. Thus, rather than continuing to ask whether intonation is a cue at all, it is worth examining when intonation is a strong cue to understanding irony. Simulating the nature of real-life situations involving irony would provide a clearer picture of how intonation interacts with various other information sources. In turn, this research would present a more thorough understanding of irony comprehension development in children (Milosky and Ford, 1997). Similarly, Nakassis and Snedeker (2002) suggested future research would benefit from examining the generalization strategy that children develop to detect and interpret irony. This would allow for a better understanding of just how broad this generalization skill is, and how it may coincide with other developmental changes.

Taking a more theoretical approach, Keenan and Quigley (1999) suggested that future studies should incorporate measures of children’s understanding of both the speaker’s intended meaning and the speaker’s communicative intention (i.e., whether the speaker meant to be understood as ironic), as this would allow for a more complete model of how irony comprehension develops. Stiles and Nadler (2013) suggested that future research should examine the interaction between intonation and context in children’s interpretation of irony to gain a deeper understanding of these processes. They further suggested the incorporation of other, less-studied cues such as speaker familiarity and facial expression.

We have reviewed only one study that highlights the importance of facial expression as a cue to children’s irony understanding (i.e., Yu et al., 2024) and the research field would certainly benefit from future research on this topic. Furthermore, Zajączkowska (2016) recommended that future researchers conduct experiments examining the effects of intonation on more complex forms of irony than sarcasm (e.g., hyperbole or understatement) to provide a more complete picture of how intonation is used as a cue in irony comprehension. We agree with the need for more breadth in figurative language forms in future research, and we additionally suggest that more research needs to be done with children who speak languages other than English. Finally, future research is needed to study online measures in this field, given that eye tracking methods and eye gaze latencies can give us a more detailed examination of how children consider intonation when deciphering a speaker’s meaning and intentions.

## 6 Conclusion

We suggest that researchers carefully consider the task demands of methods when planning future studies on children’s consideration of intonation as a cue to verbal irony, and we suggest that online methods can provide valuable information regarding the cognitive processes of verbal irony interpretation because they pose the lowest demands on children. In future studies, researchers need to acoustically analyze directional F0 changes in their intonation stimuli, include child participants who speak languages other than English, and make direct comparisons across languages, especially tonal and non-tonal ones.

## References

[ref1] Ackerman B. P. (1983). Form and function in children's understanding of ironic utterances. J. Exp. Child Psychol. 35, 487–508. doi: 10.1016/0022-0965(83)90023-1

[ref2] Aguert M. (2021). Paraverbal expression of verbal irony: vocal cues matter and facial cues even more. J. Nonverb. Behav. 46, 45–70. doi: 10.1007/s10919-021-00385-z

[ref3] Aguert M. Laval V. Le Bigot L. Bernicot J. (2010). Understanding expressive speech acts: the role of prosody and situational context in French-speaking 5- to 9-year-olds. J. Speech Lang. Hear. Res. 53, 1629–1641. doi: 10.1044/1092-4388(2010/08-0078), PMID: 20705750

[ref4] Averbeck J. M. Hample D. (2008). Ironic message production: how and why we produce ironic messages. Commun. Monogr. 75, 396–410. doi: 10.1080/03637750802512389

[ref5] Banasik-Jemielniak N. Bokus B. (2019). Children’s comprehension of irony: studies on polish-speaking preschoolers. J. Psycholinguist. Res. 48, 1217–1240. doi: 10.1007/s10936-019-09654-x, PMID: 31312955 PMC6744549

[ref6] Banasik-Jemielniak N. Kałowski P. (2022). Socio-cultural and individual factors in verbal irony use and understanding: what we know, what we don’t know, what we want to know. Rev. Commun. Res. 10, 80–113. doi: 10.12840/issn.2255-4165.036

[ref7] Bara B. G. (2010). Cognitive pragmatics: the mental processes of communication. 1st Edn. Cambridge, MA: MIT Press.

[ref8] Best C. T. (2019). The diversity of tone languages and the roles of pitch variation in non-tone languages: considerations for tone perception research. Front. Psychol. 10:364. doi: 10.3389/fpsyg.2019.00364, PMID: 30863341 PMC6399451

[ref9] Bettelli G. Giustolisi B. Panzeri F. (2024). Cross-linguistic recognition of irony through visual and acoustic cues. J. Psycholinguist. Res. 53:73. doi: 10.1007/s10936-024-10111-7, PMID: 39548017

[ref10] Blasko D. G. Kazmerski V. A. Dawood S. S. (2021). Saying what you don’t mean: a cross-cultural study of perceptions of sarcasm. Can. J. Exp. Psychol. 75, 114–119. doi: 10.1037/cep0000258, PMID: 34124932

[ref11] Bosco F. M. Angeleri R. Colle L. Katiusci S. Bara B. G. (2013). Communicative abilities in children: an assessment through different phenomena and expressive means. J. Child Lang. 40, 741–778. doi: 10.1017/S0305000913000081 23651672

[ref12] Burgers C. van Mulken M. Schellens P. J. (2011). Finding irony: an introduction of the verbal irony procedure (VIP). Metaphor Symbol. 26, 186–205. doi: 10.1080/10926488.2011.583194

[ref13] Capelli C. A. Nakagawa N. Madden C. M. (1990). How children understand sarcasm: the role of context and intonation. Child Dev. 61, 1824–1841. doi: 10.1111/j.1467-8624.1990.tb03568.x

[ref14] Cassels T. G. Birch S. A. J. (2014). Comparisons of an open-ended vs. forced-choice ‘mind reading’ task: implications for measuring perspective-taking and emotion recognition. PLoS One 9, 1–20. doi: 10.1371/journal.,pone.0093653, PMID: 25474645 PMC4256375

[ref15] Cheang H. S. Pell M. D. (2008). The sound of sarcasm. Speech Comm. 50, 366–381. doi: 10.1016/j.specom.2007.11.003

[ref16] Climie E. A. Pexman P. M. (2008). Eye gaze provides a window on children's understanding of verbal irony. J. Cogn. Dev. 9, 257–285. doi: 10.1080/15248370802247939

[ref17] Creusere M. A. (2000). A developmental test of theoretical perspectives on the understanding of verbal irony: children’s recognition of allusion and pragmatic insincerity. Metaphor Symbol. 15, 29–45. doi: 10.1207/S15327868MS151&2_3

[ref18] de Groot A. Kaplan J. Rosenblatt E. Dews S. Winner E. (1995). Understanding versus discriminating nonliteral utterances: evidence for a dissociation. Metaphor Symbol. Act. 10, 255–273. doi: 10.1207/s15327868ms1004_2

[ref19] Dews S. Winner E. Kaplan J. Rosenblatt E. Hunt M. Lim K.. (1996). Children's understanding of the meaning and functions of verbal irony. Child Dev. 67, 3071–3085. doi: 10.2307/1131767, PMID: 9071771

[ref20] Falkum I. L. Köder F. (2024). “Investigating irony comprehension in children: methods, challenges, and ways forward” in Studying verbal irony and sarcasm: Methodological perspectives from communication studies and beyond. eds. Banasik-Jemielniak N. Kałowski P. Zajączkowska M. (Cham: Palgrave Macmillan).

[ref21] Fuchs J. (2023). 40 years of research into children’s irony comprehension. Pragmat. Cogn. 30, 1–30. doi: 10.1075/pc.22015.fuc

[ref22] Glenwright M. Parackel J. Cheung K. R. J. Nilsen E. (2013). Intonation influences how children and adults interpret sarcasm. J. Child Lang. 41, 472–484. doi: 10.1017/S0305000912000773 23534818

[ref23] Glenwright M. Scott R. M. Bilevicius E. Pronovost M. Hanlon-Dearman A. (2021). Children with autism spectrum disorder can attribute false beliefs in a spontaneous-response preferential-looking task. Front. Commun. 6, 1–12. doi: 10.3389/fcomm.2021.669985

[ref24] González-Fuente S. Prieto P. Noveck I. (2016). A fine-grained analysis of the acoustic cues involved in verbal irony recognition in French. Boston: International Speech Communication Association.

[ref25] Hancock J. T. Dunham P. J. Purdy K. (2000). Children’s comprehension of critical and complimentary forms of verbal irony. J. Cogn. Dev. 1, 227–248. doi: 10.1207/S15327647JCD010204

[ref26] Hill L. J. B. Coats R. O. Mushtaq F. Williams J. J. H. Aucott L. S. Mon-Williams M. (2016). Moving to capture children’s attention: developing a methodology for measuring visuomotor attention. PLoS One 11:e0159543. doi: 10.1371/journal.,pone.0159543, PMID: 27434198 PMC4951138

[ref27] Holtgraves T. (1997). Styles of language use: individual and cultural variability in conversational indirectness. J. Pers. Soc. Psychol. 73, 624–637. doi: 10.1037//0022-3514.73.3.624

[ref28] Jorgensen J. (1996). The functions of sarcastic irony in speech. J. Pragmat. 26, 613–634. doi: 10.1016/0378-2166(95)00067-4

[ref29] Keenan T. R. Quigley K. (1999). Do young children use echoic information in their comprehension of sarcastic speech? A test of echoic mention theory. Br. J. Dev. Psychol. 17, 83–96. doi: 10.1348/026151099165168

[ref30] Köder F. Falkum I. L. (2021). Irony and perspective-taking in children: the roles of norm violations and tone of voice. Front. Psychol. 12:624604. doi: 10.3389/fpsyg.2021.624604, PMID: 34149510 PMC8209259

[ref31] Kreuz R. J. Glucksberg S. (1989). How to be sarcastic: the echoic reminder theory of verbal irony. J. Exp. Psychol. Gen. 118, 374–386. doi: 10.1037/0096-3445.118.4.374

[ref32] Kumon-Nakamura S. Glucksberg S. Brown M. (1995). How about another piece of pie: the allusional pretense theory of discourse irony. J. Exp. Psychol. Gen. 124, 3–21. doi: 10.1037/0096-3445.124.1.3, PMID: 7897341

[ref33] Laval V. Bert-Erboul A. (2005). French-speaking children’s understanding of sarcasm: the role of intonation and context. J. Speech Lang. Hear. Res. 48, 610–620. doi: 10.1044/1092-4388(2005/042), PMID: 16197276

[ref34] Li J. P. Law T. Lam G. Y. To C. K. (2013). Role of sentence-final particles and prosody in irony comprehension in Cantonese-speaking children with and without autism spectrum disorders. Clin. Linguist. Phon. 27, 18–32. doi: 10.3109/02699206.2012.734893, PMID: 23237415

[ref35] Milosavljevic A. (2024). “Experiments on the development of irony: walking through a methodological maze” in Studying verbal irony and sarcasm: Methodological perspectives from communication studies and beyond. eds. Banasik-Jemielniak N. Kałowski P. Zajączkowska M. (Cham: Palgrave Macmillan).

[ref36] Milosky L. M. Ford J. A. (1997). The role of prosody in children’s inferences of ironic intent. Discourse Process. 23, 47–61. doi: 10.1080/01638539709544981

[ref37] Nakassis C. Snedeker J. (2002). Beyond sarcasm: intonation and context as relational cues in children's recognition of irony. In: Proceedings of the 26th Boston University Conference on Language Development. Somerville: Cascadilla Press.

[ref38] Olkoniemi H. Häikiö T. Merinen M. Manninen J. Laine M. Pexman P. M. (2025). Learning irony in school: effects of metapragmatic training. J. Child Lang. 52, [online] 18 Feb, 1–22. doi: 10.1017/S0305000925000054, PMID: 39963840

[ref39] Pexman P. M. (2008). It’s fascinating research. Curr. Dir. Psychol. Sci. 17, 286–290. doi: 10.1111/j.1467-8721.2008.00591.x

[ref40] Pexman P. M. (2023). “Irony and thought: developmental insights” in The Cambridge handbook of irony and thought. eds. Gibbs R. W. Jr. Colston H. L. (Cambridge: Cambridge University Press). (Cambridge Handbooks in Psychology)

[ref41] Rattray C. Tolmie A. (2008). Young children's detection and decoding of ironic intonation. Psychol. Lang. Commun. 12, 29–54. doi: 10.2478/v10057-008-0002-1

[ref42] Rockwell P. (2000). Lower, slower, louder: vocal cues of sarcasm. J. Psycholinguist. Res. 29, 483–495.

[ref43] Rockwell P. Theriot E. M. (2001). Culture, gender, and gender mix in encoders of sarcasm: a self-assessment analysis. Commun. Res. Rep. 18, 44–52. doi: 10.1080/08824090109384781

[ref44] Scott R. M. Baillargeon R. (2009). Which penguin is this? Attributing false beliefs about object identity at 18 months. Child Dev. 80, 1172–1196. doi: 10.1111/j.1467-8624.2009.01324.x, PMID: 19630901 PMC2965529

[ref45] Sperber D. (1984). Verbal irony: pretense or echoic mention? J. Exp. Psychol. Gen. 113, 130–136. doi: 10.1037//0096-3445.113.1.130

[ref46] Stiles D. Nadler L. (2013). Sarcasm recognition in children with hearing loss: the role of context and intonation. J. Educ. Audiol. 19, 3–11.

[ref47] Whalen J. M. Doyle A. Pexman P. M. (2020). Sarcasm between siblings: children’s use of relationship information in processing ironic remarks. J. Pragmat. 156, 149–159. doi: 10.1016/j.pragma.2019.05.005

[ref48] Wilson D. Sperber D. (2012). “Explaining irony” in Meaning and relevance. eds. Wilson D. Sperber D. (Cambridge, MA: Cambridge University Press).

[ref49] Winner E. Windmueller G. Rosenblatt E. Bosco L. Best E. Gardner H. (1987). Making sense of literal and nonliteral falsehood. Metaphor Symbol. Act. 2, 13–32. doi: 10.1207/s15327868ms0201_2

[ref50] Yu Q. Li H. Li S. Tang P. (2024). Prosodic and visual cues facilitate irony comprehension by mandarin-speaking children with cochlear implants. J. Speech Lang. Hear. Res. 67, 2172–2190. doi: 10.1044/2024_JSLHR-23-00701, PMID: 38820233

[ref51] Zajączkowska M. K. (2016). Influence of voice intonation on understanding irony by polish-speaking preschool children. Psychol. Lang. Commun. 20, 278–291. doi: 10.1515/plc-2016-0017

